# RETRACTED: Li et al. Protective Effects of Astaxanthin Supplementation Against Ultraviolet-Induced Photoaging in Hairless Mice. *Biomedicines* 2020, *8*, 18

**DOI:** 10.3390/biomedicines14051148

**Published:** 2026-05-19

**Authors:** Xing Li, Tomohiro Matsumoto, Miho Takuwa, Mahmood Saeed Ebrahim Shaiku Ali, Takumi Hirabashi, Hiroyo Kondo, Hidemi Fujino

**Affiliations:** Department of Rehabilitation Science, Kobe University Graduate School of Health Sciences, 7-10-2 Tomogaoka, Suma-ku, Kobe 654-0142, Hyogo, Japan; lixing3633@gmail.com (X.L.); tomo08047777@gmail.com (T.M.); miho.takuwa@gmail.com (M.T.); mahmood_6195@hotmail.com (M.S.E.S.A.); hirabayashitkm1@gmail.com (T.H.); hiroyokondo@gmail.com (H.K.)

The journal has retracted the article “Protective Effects of Astaxanthin Supplementation against Ultraviolet-Induced Photoaging in Hairless Mice” [1] cited above.

Following publication, concerns were brought to the attention of the Editorial Office regarding the integrity of a figure presented in this publication [1].

In accordance with standard journal procedures, the Editorial Office and Editorial Board conducted an investigation that confirmed inappropriate editing and partial overlap in Figure 2 of this publication [1]. While the authors cooperated during the investigation, they were unable to provide original images or materials that met the Editorial Board’s requirement to resolve these concerns. As a result, the Editorial Board has lost confidence in the reliability of the findings and has decided to retract this publication, as per MDPI’s retraction policy (https://www.mdpi.com/ethics#_bookmark30, accessed on 2 February 2026).

This retraction was approved by the Editor-in-Chief of the *Biomedicines* journal.

The authors have been informed of this retraction and have indicated their disagreement with this decision.
